# A COMPARATIVE STUDY OF INTEGRATED LONG-TERM CARE SERVICES IN TAIPEI CITY AND NINGBO CITY

**DOI:** 10.1093/geroni/igae098.3584

**Published:** 2024-12-31

**Authors:** Huan Liu, Hong Mi

**Affiliations:** NingBo University of Technology, NingBo, Zhejiang, China (People’s Republic); Zhejiang University, Hangzhou, Zhejiang, China (People’s Republic)

## Abstract

This study aims to compare the integrated long-term care service models in Taipei and Ningbo, exploring their similarities, differences, and experiences for mutual learning. Through literature review and field research, it was found that Taiwan’s long-term care system has advantages such as comprehensive coverage, diversified services, professional teams, smart technology applications, community participation, policy support, and a supportive social and cultural environment. On the other hand, Ningbo’s integrated long-term care services also have unique features in terms of service content and format, emphasizing family care, promoting the integration of medical care and older people care, and developing community rehabilitation services. The comparative study reveals that Taiwan’s long-term care system is more mature and comprehensive, with higher service quality and coverage, while Ningbo has certain advantages in the integration of medical care and older people care, as well as in community rehabilitation services. Based on this, the study proposes recommendations for the complementary development of long-term care service models in Taipei and Ningbo, including strengthening policy coordination, promoting inter-departmental cooperation, and enhancing service quality and efficiency, aiming to provide reference for the improvement and development of long-term care services in both regions.

